# Correlation Between Depression and Quality of Life Among Patients With Parkinson’s Disease: An Analytical Cross-Sectional Study

**DOI:** 10.7759/cureus.54736

**Published:** 2024-02-23

**Authors:** Priya Sujith, Porkodi Arjunan, Thomas Iype, Venkatesh Natarajan

**Affiliations:** 1 Nursing, Government College of Nursing, Thiruvananthapuram, IND; 2 Nursing, Sri Ramachandra Institute of Higher Education and Research, Chennai, IND; 3 Medical Surgical Nursing, Sri Ramachandra Institute of Higher Education and Research, Chennai, IND; 4 Neurology, Medical College Thiruvananthapuram, Thiruvananthapuram, IND; 5 Physiotherapy, Sri Ramachandra Institute of Higher Education and Research, Chennai, IND

**Keywords:** bradykinesia, movement disorder, neuro psychiatric problems, updrs, non-motor symptoms, motor symptoms, anxiety, depression, parkinson's, quality of life

## Abstract

Introduction

Parkinson’s disease (PD) is a progressive complex degenerative disorder characterised by several motor and non-motor symptoms that result in disability and deterioration of the patient's quality of life (QOL). Depression is the most common non-motor symptom that may severely alter the QOL. The objective of this study was to examine the correlation between depression and QOL among patients with PD who received treatment from a movement disorder clinic of a tertiary care teaching hospital in South India.

Methods

This was an analytical cross-sectional study conducted among 220 PD patients who received treatment from a movement disorder clinic of a tertiary care teaching hospital in South India. The participants aged between 40 and 80 years, who can comprehend Malayalam or English and were clinically diagnosed with PD according to United Kingdom PD Society Brain Bank criteria were included in the study. Depression was assessed using the Hospital Anxiety and Depression Scale, motor function using the Movement Disorder Society Unified Parkinson’s Disease Rating Scale Part III, and the quality of life was assessed using the Parkinson’s Disease Questionnaire 39.

Results

The results of this study showed that there was a significant positive correlation between depression and QOL (r=0.699, p<0.01) among patients with PD who received treatment from a tertiary care teaching hospital. The correlation with domains of QOL also identified that depression was significantly correlated with all domains of QOL and more to the emotional domain of QOL (r=0.799, p<0.01).

Conclusion

Depression is the most common neuropsychiatric condition in PD and the most important determinant of QOL. Depression may occur at any stage of PD and can significantly impact the QOL of patients and their caregivers. Hence it should be recognized early and managed by pharmacological and nonpharmacological measures to improve the QOL.

## Introduction

The ageing population is growing globally, and the burden of neurological diseases is increasing rapidly posing a challenge to the sustainability of health systems, especially the low and middle-income countries. Parkinson's disease (PD) is the most common progressive degenerative neurological disease that leads to significant disability and poor quality of life. Globally, 6.1 million individuals had PD in 2016. There was a greater increase in the number of individuals with PD from 1990 to 2016 (2.4 times higher than in 1990). In 1990, 2.5 million people had PD. Globally PD caused 211296 deaths in 2016. [1]

In India, the prevalence of PD was 55 (95% CI: 46-66) per 100000 population in 2019. The percentage change in the crude prevalence of PD in India from 1990-2019 was 105.9% (95% CI: 97.2-115.0). Prevalence increased notably in the older age groups particularly in those older than 50 years, both in males and females. In males, the prevalence is 69.9 (95% CI: 51.1-92.5), and in females 53.2 (95% CI: 37.6-70.7). In India, an estimated 45300 people died due to PD in 2019 [2].

PD can cause both motor and non-motor symptoms. The cardinal signs of PD are movement-related and include bradykinesia, tremors, muscular rigidity, and postural instability. Non-motor symptoms of PD are cognitive and behavioural problems, sleep problems, emotional problems, depression, anxiety, constipation, difficulties in coordination and speech, severe fatigue, orthostatic hypotension, and pain [3]. In particular, the most common neuropsychiatric and disabling condition is depression which may occur at any stage of PD, including the prodromal stage, and may severely alter the quality of life (QOL) of both patients and their caregivers. The pathological mechanism of PD depression starts in the prodromal state and the progression of dopaminergic and non-dopaminergic pathology during PD explains the variable expression and outcome of depression over time [4]. 

Several socio-demographic and clinical factors may contribute to poor QOL in PD patients. Both motor and non-motor symptoms, psychosocial dysfunctions, and psychiatric comorbidities are common in PD patients, which could lower their QOL. Motor symptoms such as walking abnormalities and postural instability contribute to the deterioration of the QOL in PD patients. Worsening motor functions, advanced stage of disease, and higher prevalence of falls may deteriorate the psychological aspects of QOL. There is a negative effect of depression on the QOL of patients with PD and depression is the most important determinant of QOL [5-11].

During the early stages of PD, levodopa, the precursor of dopamine, increases the duration of time the patients remain independent and employable and improve the QOL by acting on the predominant motor symptoms. However, the use of the same drug in a higher dose is less effective during the late stage and its side effects further deteriorate patients' QOL [12]. As the symptoms of both PD and depression are overlapped depression is often underdiagnosed and this may affect the QOL of patients with PD. [11]. Since PD is incurable, it is highly important to establish the most effective way to improve the QOL. Hence, accurate diagnosis of depression and motor and non-motor symptoms in PD, at each stage, is critical to improve QOL [4]. We were interested in determining the relationship between depression and QOL among patients with PD who received treatment from a tertiary care teaching hospital. Hence, we hypothesized that there is a statistically significant relationship between depression and QOL among patients with PD who received treatment from a tertiary care teaching hospital. Hence this study aimed to examine the correlation between depression and QOL among patients with PD attending the movement disorder clinic of a tertiary care teaching hospital in South India.

## Materials and methods

Study design

The research design adopted for this study was an Analytical Cross-sectional design. This design was selected to find out the correlation between depression and the Quality of Life of patients with PD. The study setting was the movement disorder clinic of a tertiary care teaching hospital in South India. The population of this study was patients diagnosed with PD.

Ethical considerations

Ethical approval and study setting permission were obtained before data collection. The study was approved by the Institutional Ethics Committee of the Government College of Nursing, Thiruvananthapuram (IEC-N1/19/NOV/83).

Study criteria

The inclusion criteria were male and female patients clinically diagnosed with PD according to United Kingdom PD Society Brain Bank criteria [13], aged between 40 and 80 years, and who can comprehend Malayalam or English. Exclusion criteria were duration of PD less than three months, known psychiatric diagnosis, mental retardation, visual/hearing impairment, severe dyskinesia, and severe medical conditions that affect the QOL.

Procedure

Data was collected from the PD patients who attended the movement disorder clinic of a tertiary care teaching hospital and who fulfilled the inclusion criteria. The participants were selected consecutively after obtaining written informed consent. The data collection period was from December 2021 to February 2023. The techniques used for collecting data were interview technique, self-reports, and record review.

Assessments

The instruments used for data collection were as follows:

Structured Questionnaire to Assess Socio-Demographic and Clinical Data

It includes age, gender, education, marital status, living arrangements, duration of PD, duration of treatment with Levodopa, stage of PD, and co-morbidities like diabetes mellitus, hypertension, heart disease, respiratory disease, etc. The Hoehn and Yahr scale was used to stage the PD based on the severity of the disease. There are five stages of progressive impairment and disability. PD severity is described as a progression from stage 1 with unilateral motor involvement to stage 5 with confinement to a bed or wheelchair [14]

Hospital Anxiety and Depression Scale (HADS)

This was used to collect data regarding depression and anxiety. HADS consists of 14 items, seven items for depression and seven items for anxiety. The minimum score for each item is 0 and the maximum is 3. A score of >8 represents either depression or anxiety [15]. The reliability of HADS in patients with PD was good. (Cronbach alpha 0.88) [16].

Movement Disorder Society Unified Parkinson’s Disease Rating Scale Part III (MDS UPDRS Part III)

The severity of motor symptoms was assessed using this tool. It consists of 18 items. Each question is anchored with five responses 0=normal, 1=slight, 2=mild, 3= moderate, and 4= severe. As the score increases motor function decreases [17, 18].

Parkinson’s Disease Questionnaire (PDQ 39)

This tool was used to assess the QOL of patients with PD. This is a disease-specific 39-item questionnaire grouped into eight sub-scales including mobility, Activities of Daily Living (ADL), emotional well-being, stigma, social support, cognition, communication, and bodily discomfort. The responses are scored from 0 (never) to 4 (always). A Summary Index (PDQ 39 SI) and the weighted percentage of the problem’s severity were calculated based on the scoring guideline. The original scores were transformed to a scale from 0 to 100. The higher score represents the worse QOL. A validated Malayalam version of PDQ 39 was used in this study [19].

Sample size calculation

The sample size was calculated based on the prevalence rate of depression of 31.25% [5], a precision of 80%, and a standard value (Z) of 1.96. The total sample size was 220.

Statistical analysis

The SPSS Statistics version 20.0 (IBM Corp. Released 2011. IBM SPSS Statistics for Windows, Version 20.0. Armonk, NY: IBM Corp) was used for all statistical analysis. Quantitative variables were expressed as median and inter-quartile range and qualitative variables as percentages. The correlation between depression, motor function, and QOL was assessed using Karl Pearson’s correlation coefficient. The variables that were not normally distributed were transformed into a normal distribution by the square root method. A value of p<0.05 was considered statistically significant.

## Results

A total of 220 patients were enrolled in the study. Among these patients, 50.5% were males, married 83.6%, non-smokers 92.3%, and non-alcoholic 96.4%. The majority of the participants had no family history of PD (90%), 43.6% had no co-morbidities and 49.5% belonged to Hoehn and Yahr stage 2. The mean age and education of the participants in years were 60.36 (7.86) and 8.86 (3.90) respectively.

Table 1 shows the clinical data of our participants. Since the data was not normally distributed we presented with median and interquartile range (IQR). The median and IQR on disease duration of PD and treatment duration with levodopa were 4(3.0-7.0) and 4(2.0-7.0) respectively. The median MDS UPDRS Part III score of motor examination was 30 (18.25-43.0) and the median anxiety and depression scores using HADS were 5(2.25-7.0) and 4(2.0-8.0) respectively. The median PDQ 39 SI was 16.50(9.0-26.75) and the median and IQR on various domains of PDQ 39 were mobility 13(3.0-30.0), ADL 15(4.0-33.0), emotional well-being 17(4.0-33.0), stigma 6(0.00-25.0), cognition 16(6.0-31.0), communication 8(0.00-17.0) and discomfort 33(17.0-42.0) respectively. 

**Table 1:** Median, IQR, Minimum and Maximum values of clinical variables IQR: interquartile range, MDS UPDRS: Movement Disorder Society unified Parkinson's disease rating scale, PDQ 39 SI: Parkinson's disease questionnaire 39 summary index, ADL: activities of daily living

Variables	Median (IQR)	Minimum	Maximum
Disease duration in years	4.0(3.0-7.0)	0.25	15.0
Treatment duration with Levodopa in years	4.0(2.0-7.0)	0.25	15.0
MDS UPDRS Score	30.0(18.25-43.0	6	89
Anxiety	5.0(2.25-7.0)	0	17
Depression	4.0 ()2.0-8.0	0	16
PDQ 39 SI	16.50 (9.00-26.75)	1	74
PDQ Domains Mobility	13.0(3.0-30.0)	0	100
ADL	15.0 (4.0-33.0)	0	100
Emotions	17.0(4.0-33.0)	0	83
Stigma	6.0 (0.0-25.0)	0	88
Social support	0.0 (0.0-8.0)	0	75
Cognition	16.0 (6.0-31.0)	0	88
Communication	8.0 (0.0-17.0)	0	100
Discomfort	33.0 (17.0-42.0)	0	100

Correlation between anxiety, depression, motor function, and QOL among patients with PD

**Figure 1:**
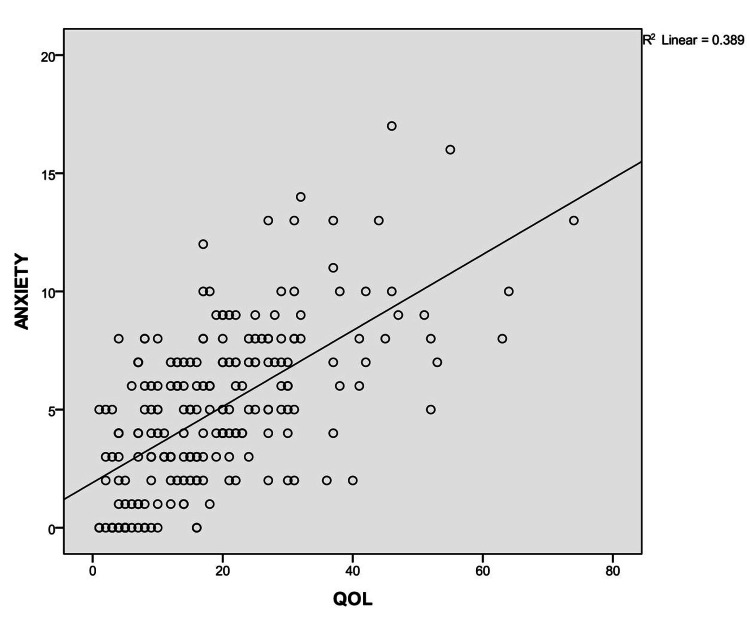
Correlation between anxiety and QOL among patients with Parkinson's disease QOL: quality of life, R: correlation coefficient

Figure 1 shows a positive correlation between anxiety and QOL among patients with PD (r=0.624, p<0.01).

**Figure 2:**
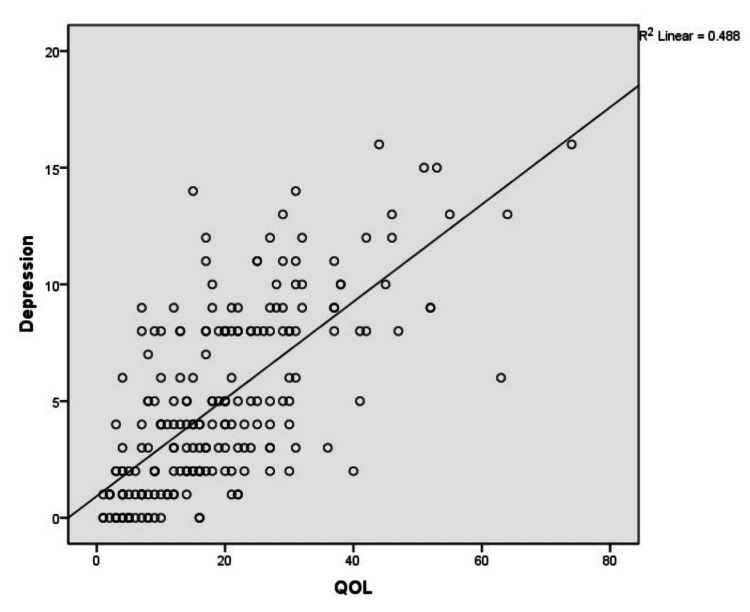
Correlation between depression and QOL among patients with PD QOL: quality of life, R: correlation coefficient

Figure 2 shows a positive correlation between depression and QOL among patients with PD (r=0.699, p<0.01).

**Figure 3:**
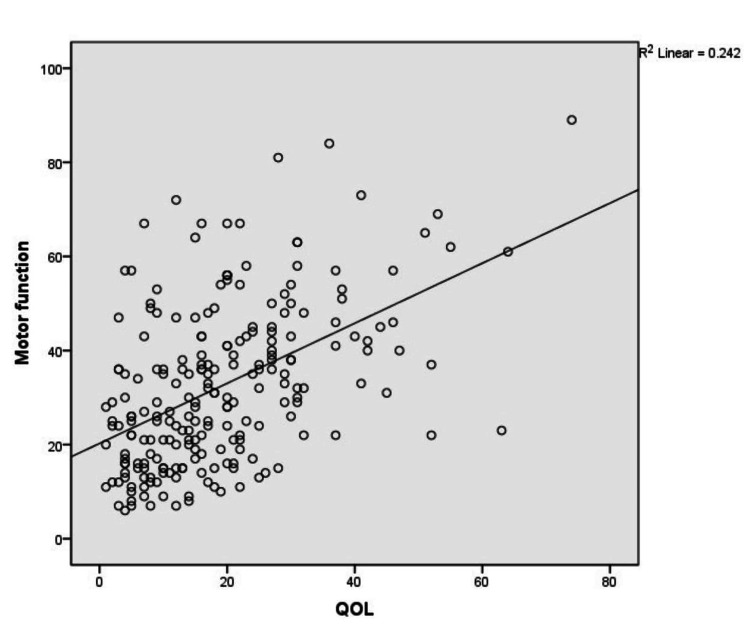
Correlation between motor function and QOL among patients with Parkinson's disease QOL: quality of life, R: correlation coefficient

Figure 3 shows a positive correlation between motor function and QOL among patients with PD (r=0.492, p<0.01).

Table 2 shows the correlation between depression, anxiety, motor function, and domains of QOL. Depression, anxiety, and motor function were significantly correlated with all domains of QOL.

**Table 2:** Correlation between anxiety, depression, motor function and domains of QOL ADL: activities of daily living; QOL: quality of life

Variables		Mobility	ADL	Emotions	Stigma	Social support	Cognition	Communication	Bodily Discomfort
Anxiety	Pearson’s correlation	0.431	0.391	0.742	0.392	0.299	0.348	0.487	0.314
	Significant (2- tailed)	P<0.01	P<0.01	P<0.01	P<0.01	P<0.01	P<0.01	P<0.01	P<0.01
Depression	Pearson’s correlation	0.470	0.440	0.799	0.433	0.336	0.395	0.510	0.333
	Significant (2- tailed)	P<0.01	P<0.01	P<0.01	P<0.01	P<0.01	P<0.01	P<0.01	P<0.01
Motor function	Pearson’s correlation	0.399	0.436	0.263	0.251	0.278	0.299	0.404	0.212
	Significant (2- tailed)	P<0.01	P<0.01	P<0.01	P<0.01	P<0.01	P<0.01	P<0.01	P<0.01

Correlation With QOL Domain Emotional Wellbeing

Depression had a significant positive correlation with QOL domain emotional well-being (r=0.799, p<0.01). Anxiety and motor function were significantly correlated with the emotional well-being domain of QOL (r=0.742, p<0.01 and r=0.263, p<0.01).

Correlation With QOL Domain Communication

Depression had a significant positive correlation with the QOL domain communication (r=0.510, p<0.01). Anxiety and motor function were significantly correlated with the QOL domain communication (r=0.487, p<0.01 and r=0.404, p<0.01).

Correlation With QOL Domain Mobility

Depression was significantly correlated to the QOL domain mobility (r=0.470, p<0.01). There was a significant positive correlation between the mobility domain of QOL and anxiety and motor function (r=0.431, p<0.01 and r=0.399, p<0.01).

Correlation With QOL Domain ADL

There was a significant positive correlation between depression and the QOL domain ADL ( r=0.440, p<0.01). Motor function and anxiety were significantly correlated with the ADL domain of QOL (r=0.436, p<0.01 and r=0.391, p<0.01).

Correlation With QOL Domain Stigma

The correlation between depression and QOL domain stigma shows a significant positive correlation ( r= 0.433, p<0.01). There was a significant positive correlation between QOL domain stigma and anxiety and motor function ( r=0.392, p<0.01 and r=0.251, p< 0.01).

Correlation With QOL Domain Cognition

Depression was significantly correlated with the QOL domain, cognition (r=0.395, p<0.01). Anxiety and motor function were significantly correlated with the cognition domain of QOL (r=0.348, p<0.01 and r=0.299, p<0.01)

Correlation With QOL Domain Social Support

There was a significant positive correlation between depression and QOL domain social support (r=0.336, p<0.01). Anxiety and motor function were significantly correlated with QOL domain social support (r=0.299, p<0.01 and r=0.278, p<0.01).

Correlation With QOL Domain Bodily Discomfort

There was a significant positive correlation between depression and QOL domain bodily discomfort (r=0.333, p<0.01). Anxiety and motor function were significantly correlated with QOL domain bodily discomfort (r=0.314, p<0.01 and r=0.212, p<0.01).

## Discussion

Depression is reported to be common in PD. Depression is the most common neuropsychiatric and disabling condition which may occur at any stage of PD. There is a negative effect of depression on the QOL of patients with PD. The QOL was significantly worse in PD patients with depression than those without depression. Since PD is incurable it is highly important to improve the QOL of patients with PD [ 4,5,10]. Hence we hypothesised that there is a statistically significant relationship between depression and QOL among PD patients who received treatment from a tertiary care teaching hospital. Our results also concluded that there was a statistically significant positive correlation between depression and QOL among patients with PD who received treatment from a tertiary care teaching hospital. The finding of our study was supported by Lacy et al. where prolonged disease duration, severe depressive symptoms, and motor symptoms significantly correlate with poor QOL even though these patients were treated in a high-quality academic movement clinic [20].

In most of the studies, depression is the most common non-motor symptom associated with poor QOL than motor symptoms [21-22]. In our study also depression is positively correlated with poor QOL (r=0.699, p<0.01) than motor symptoms among patients with PD (r=0.492, p<0.01). In the present study, there was a strong positive correlation between anxiety and poor QOL (r=0.624, p<0.01). This is consistent with the findings from another study where anxiety was the significant determinant of Health-related Quality of Life among PD patients [23].

In the present study, there was a positive correlation between depression and all QOL domains (emotional well-being (r=0.799, p<0.01), communication (r=0.510, p<0.01), mobility (r =0.470, p< 0.01), ADL (r=0.440, p<0.01), stigma (r=0.433, p<0.01). These findings were supported by the study by Su W et al where depression and anxiety correlate with all domains of Quality of life [24].

The correlation between anxiety and QOL domain shows a positive correlation between anxiety and all domains of QOL (emotional well-being (r=0.742, p< 0.01), communication (r=0.487, p<0.01), mobility (r =0.431, p< 0.01), stigma (r=0.392, p<0.01), ADL (r=0.391, p<0.01). Like Chuquilín-Arista et al and Upneja et al also reported a significant linear relationship between anxiety and QOL [23, 25].

The correlation between motor function (UPDRS part III) and QOL domains shows a positive correlation in all domains of QOL( ADL (r=0.436, p<0.01), communication (r=0.404, p<0.01, mobility (r=0.399, p<0.01). These findings were in line with another study where severe motor symptoms correlated most strongly with PDQ 39 domains impaired mobility and ADL [20].

We identified that there was a statistically significant positive correlation between depression and QOL among patients with PD. Anxiety and motor function were also positively correlated with the QOL of PD patients. The correlation with domains of QOL identified that depression, anxiety, and motor function correlated significantly to all domains of QOL but depression contributed strongly to the emotion domain of QOL. Like other studies, our findings also suggest that depression contributed more to QOL than motor function. [20-23, 26]

Limitations

Although our study contributes to a better understanding of the correlation between depression and QOL there are still some limitations. The methodological limitation is, that the study is cross-sectional. A well-defined longitudinal study will improve the power of the study and it will better capture the changes in QOL. The next limitation is the lack of detailed data regarding the treatment of PD like levodopa dosage, number of pills per day, and adverse effects of levodopa. This will pose a challenge in distinguishing between depression and the side effects of the drug. Since the setting of our study is a movement disorder clinic of a tertiary care teaching hospital, the majority of our samples were ambulatory and fewer were in advanced stages of disease. Hence the study findings cannot be generalised to the entire PD population. Another limitation is difficulty in making the diagnosis of depression as most of the symptoms of PD and depression are overlapped. In the present study, depression was measured using HADS instead of the Diagnostic and Statistical Manual of Mental Disorders, Fifth Edition criteria which is the gold standard for depressive disorders. However, the internal consistency and test-retest reliability of HADS are good in patients with PD (Cronbach alpha 0.88). Hence it can be used to screen depression in PD patients [16].

## Conclusions

Depression is the most common neuropsychiatric condition in PD and the most important determinant of QOL. The present study aimed to examine the correlation between depression and QOL of patients with PD. Results suggest that there is a significant positive correlation between depression and QOL in patients with PD. Depression was significantly correlated with all domains of QOL and more to the emotional well-being domain of QOL. The study highlights the need for early recognition and accurate diagnosis of depression in PD patients and the importance of pharmacological and non-pharmacological measures in the treatment of depression to improve the QOL of patients with PD.

## References

[1] (2018). Global, regional, and national burden of Parkinson's disease, 1990-2016: a systematic analysis for the Global Burden of Disease Study 2016. Lancet Neurol.

[2] (2021). The burden of neurological disorders across the states of India: the Global Burden of Disease Study 1990-2019. Lancet Glob Health.

[3] Opara J, Brola W, Leonardi M, Błaszczyk B (2012). Quality of life in Parkinso's Disease. J Med Life.

[4] Prange S, Klinger H, Laurencin C, Danaila T, Thobois S (2022). Depression in patients with Parkinson's Disease: current understanding of its neurobiology and implications for treatment. Drugs Aging.

[5] Khedr EM, Abdelrahman AA, Elserogy Y, Zaki AF, Gamea A (2020). Depression and anxiety among patients with Parkinson’s disease: frequency, risk factors and impact on quality of life. Egypt J Neurol Psych Neurosurg.

[6] Santos García D, de Deus Fonticoba T, Cores C (2021). Predictors of clinically significant quality of life impairment in Parkinson's disease. NPJ Parkinsons Dis.

[7] Al-Khammash N, Al-Jabri N, Albishi A (2023). Quality of life in patients with Parkinson's Disease: a cross-sectional study. Cureus.

[8] Zhao N, Yang Y, Zhang L (2020). Quality of life in Parkinson's disease: a systematic review and meta‐analysis of comparative studies. CNS Neurosci Ther.

[9] Fan JY, Chang BL, Wu YR (2016). Relationships among depression, anxiety, sleep, and quality of life in patients with Parkinson's disease in Taiwan. Parkinsons Dis.

[10] Prakash KM, Nadkarni NV, Lye WK, Yong MH, Tan EK (2016). The impact of non-motor symptoms on the quality of life of Parkinson's disease patients: a longitudinal study. Eur J Neurol.

[11] Menon B, Nayar R, Kumar S, Cherkil S, Venkatachalam A, Surendran K, Deepak KS (2015). Parkinson's disease, depression, and quality-of-life. Indian J Psychol Med.

[12] Martinez-Martin P, Rodriguez-Blazquez C, Forjaz MJ, Kurtis MM (2015). Impact of pharmacotherapy on quality of life in patients with Parkinson's disease. CNS Drugs.

[13] Rajput DR (1993). Accuracy of clinical diagnosis of idiopathic Parkinson's disease. J Neurol Neurosurg Psych.

[14] Martinez-Martin P, Skorvanek M, Rojo-Abuin JM, Gregova Z, Stebbins GT, Goetz CG (2018). Validation study of the hoehn and yahr scale included in the MDS-UPDRS. Mov Disord.

[15] Zigmond AS, Snaith RP (1983). The hospital anxiety and depression scale. Acta Psychiatr Scand.

[16] Schrag A, Barone P, Brown RG (2007). Depression rating scales in Parkinson's disease: critique and recommendations. Mov Disord.

[17] Goetz CG, Tilley BC, Shaftman SR (2008). Movement Disorder Society-sponsored revision of the Unified Parkinson's Disease Rating Scale (MDS-UPDRS): scale presentation and clinimetric testing results. Mov Disord.

[18] Evers LJ, Krijthe JH, Meinders MJ, Bloem BR, Heskes TM (2019). Measuring Parkinson's disease over time: the real‐world within‐subject reliability of the MDS‐UPDRS. Mov Disord.

[19] Jenkinson C, Fitzpatrick R, Peto V, Greenhall R, Hyman N (1997). The Parkinson's Disease Questionnaire (PDQ-39): development and validation of a Parkinson's disease summary index score. Age Ageing.

[20] Lacy B, Piotrowski HJ, Dewey RB Jr, Husain MM (2023). Severity of depressive and motor symptoms impacts quality of life in Parkinson's disease patients at an academic movement clinic: a cross-sectional study. Clin Park Relat Disord.

[21] Yoon JE, Kim JS, Jang W (2017). Gender differences of nonmotor symptoms affecting quality of life in Parkinson disease. Neurodegener Dis.

[22] Weintraub D, Moberg PJ, Duda JE, Katz IR, Stern MB (2004). Effect of psychiatric and other nonmotor symptoms on disability in Parkinson's disease. J Am Geriatr Soc.

[23] Chuquilín-Arista F, Álvarez-Avellón T, Menéndez-González M (2021). Impact of depression and anxiety on dimensions of health-related quality of life in subjects with Parkinson’s disease enrolled in an association of patients. Brain sciences.

[24] Su W, Liu H, Jiang Y, Li S, Jin Y, Yan C, Chen H (2021). Correlation between depression and quality of life in patients with Parkinson's disease. Clin Neurol Neurosurg.

[25] Upneja A, Paul BS, Jain D, Choudhary R, Paul G (2021). Anxiety in Parkinson's disease: correlation with depression and quality of life. J Neurosci Rural Pract.

[26] He L, Lee EY, Sterling NW (2016). The key determinants to quality of life in Parkinson's Disease patients: results from the parkinson's disease biomarker program (pdbp). J Parkinsons Dis.

